# Identification of anoikis-related genes and immune infiltration characteristics in Sjögren’s syndrome based on machine learning

**DOI:** 10.3389/fmed.2025.1661259

**Published:** 2025-11-03

**Authors:** Lei Wang, Ziqi Xu, Xinpeng Zhou, Ying Liu, Mengjie Wang

**Affiliations:** 1 Department of Rheumatology, Affiliated Hospital of Shandong University of Traditional Chinese Medicine, Jinan, China; 2 Institute of Pharmacy, Shandong University of Traditional Chinese Medicine, Jinan, China

**Keywords:** Sjögren’s syndrome, anoikis, machine learning, ceRNA network, immune infiltration

## Abstract

**Objective:**

Anoikis, a recently identified type of programmed cell death analogous to apoptosis, has been implicated in the pathogenesis of Sjögren’s syndrome (SS). Although accumulating evidence indicates its involvement in modulating immune responses and contributing to SS progression, the precise role of anoikis in SS remains inadequately understood. This study aimed to explore anoikis-related genes (ARGs) and their molecular mechanisms in SS using public databases.

**Methods:**

SS datasets (GSE23117, GSE84844 and GSE12795) were retrieved from the GEO database. In total, 924 ARGs were extracted from the GeneCards and Harmonizome databases, followed by differential expression gene (DEGs) analysis and weighted gene co-expression network analysis (WGCNA). Machine learning algorithms were utilized to screen candidate biomarkers, and their diagnostic effectiveness was assessed using receiver operating characteristic (ROC) curve analysis. Concurrently, a mouse model of SS was established and validated through *in vivo* experiments. Immune cell infiltration in SS tissues was evaluated using CIBERSORT, and correlations between characteristic genes and immune cell profiles were analyzed. Potential drug candidates targeting these genes were identified using the DGIdb database. Subsequently, an lncRNA-miRNA-mRNA network associated with these genes was constructed, and preliminary experimental validation was conducted.

**Results:**

A total of 35 differentially expressed anoikis-related genes (DEARGs) were identified. GO and KEGG enrichment analyses demonstrated that DEARGs were primarily associated with inflammation, viral infections, and the necroptosis signaling pathway. Machine learning analysis pinpointed 14 feature genes, among seven were associated with cancer (*NAT1*, *BIRC3*, *EZH2*, *MAD2L1*, *ATP2A3*, *HMGA1*, and *BST2*). Given the unclear roles of *SKI* and *PRDX4* in SS, the study focused specifically on five relevant genes, *MAPK3, IL15, S100A9, IFI27*, and *CXCL10*, which were validated by *in vivo* experiments. Immune cell analysis revealed increased proportions of B cells, T cells, macrophages, and other immune cells in SS tissues. Furthermore, ceRNA and drug-gene interaction networks were established, underscoring the regulatory significance of five key miRNAs (miR-30b-5p, miR-148a-3p, miR-130a, miR-483-5p, and miR-486-3p) in SS. In addition, eight candidate drugs were identified with potential for modulating SS pathogenesis.

**Conclusion:**

This study substantiates the significant involvement of anoikis in SS and suggests that *MAPK3, IL15, S100A9, IFI27*, and *CXCL10* may serve as critical biomarkers in the inflammatory progression of SS. These genes likely mediate their effects by influencing immune cell infiltration, participating in immune regulation, and modulating inflammatory responses. Our findings offer new insights into drug selection and immunotherapeutic strategies for SS.

## 1 Introduction

Sjögren’s Syndrome (SS) is an autoimmune disorder characterized by immune cell infiltration of the lacrimal and salivary glands (1), resulting in reduced tear and saliva secretion. Clinically, SS primarily manifests as oral and ocular dryness, frequently accompanied by joint pain and fatigue. With a prevalence ranging from 0.29 to 0.77%, it is among the most common rheumatic immune diseases. Importantly, SS patients have a markedly elevated risk, 30–40 times higher than that of the general population, of developing malignant lymphoma, severely affecting both life expectancy and quality of life (2). A hallmark of SS is focal lymphocytic sialadenitis within the salivary glands, which are both the primary target organs and central to disease pathogenesis. Glandular stromal cells, including endothelial, epithelial, and fibroblast populations, play a crucial role in shaping the glandular immune microenvironment (3). However, the mechanisms underlying glandular injury in SS remain incompletely elucidated, though they clearly involve multiple functional impairments and aberrant apoptosis of glandular cells.

Anoikis, a caspase-dependent form of cell death similar to apoptosis but distinct in being triggered by cell detachment from the extracellular matrix (ECM) (4). Anoikis occurs through the disruption of integrin-mediated adhesion. This process prevents abnormal cell growth or attachment to inappropriate substrates (5). Anoikis participates in diverse physiological functions, including gland morphogenesis and the maintenance of normal epithelial tissue architecture and homeostasis (6). Its dysregulation has been implicated in tumor cell transformation and metastasis. Emerging evidence suggests that anoikis also plays a key role in modulating immune responses and may contribute to the pathogenesis of SS. Notably, SS-affected glands display ECM degradation, impaired epithelial regeneration, and progressive inflammation (7). In SS, epithelial cells of exocrine glands (e.g., salivary and lacrimal glands) exhibit reduced adhesion to the ECM due to altered ECM components and abnormal expression of integrins and other matrix receptors, leading to detachment from their normal microenvironment. Simultaneously, aberrant immune activation (e.g., T and B cells) along with oxidative and endoplasmic reticulum stress further disrupts epithelial cell-matrix connections or impairs survival signaling pathways, collectively triggering anoikis. In this process, epithelial cell apoptosis not only directly causes glandular dysfunction but also induces the release of autoantigens such as TRIM21 and La/SSB via lysosomal-associated membrane protein 3 (LAMP3), thereby exacerbating autoimmunity. This creates a vicious cycle between the inflammatory microenvironment and anoikis, further aggravating disease severity. Compared with other forms of programmed cell death, anoikis occurs more readily in SS due to the combined effects of epithelial cell-matrix dysregulation and immune system disturbances (8). However, the role of anoikis in SS pathogenesis remains poorly explored. On this basis, we hypothesize a strong association between anoikis and the development of SS.

The immune mechanisms underlying SS remain largely unknown. Identifying novel characteristic genes may provide potential targets and insights into SS etiology. In this study, we present the first comprehensive analysis of the intrinsic relationship between SS and anoikis. We perform functional enrichment analysis of differentially expressed genes (DEGs) and anoikis-related genes (ARGs) in SS, employ machine learning algorithms to identify key characteristic genes, and examine the relationships between these genes, immune cell infiltration, and regulatory networks. Our findings aim to provide a theoretical foundation and new perspectives for developing treatment strategies for SS.

## 2 Materials and methods

### 2.1 Data acquisition and preprocessing

**Figure 1:**
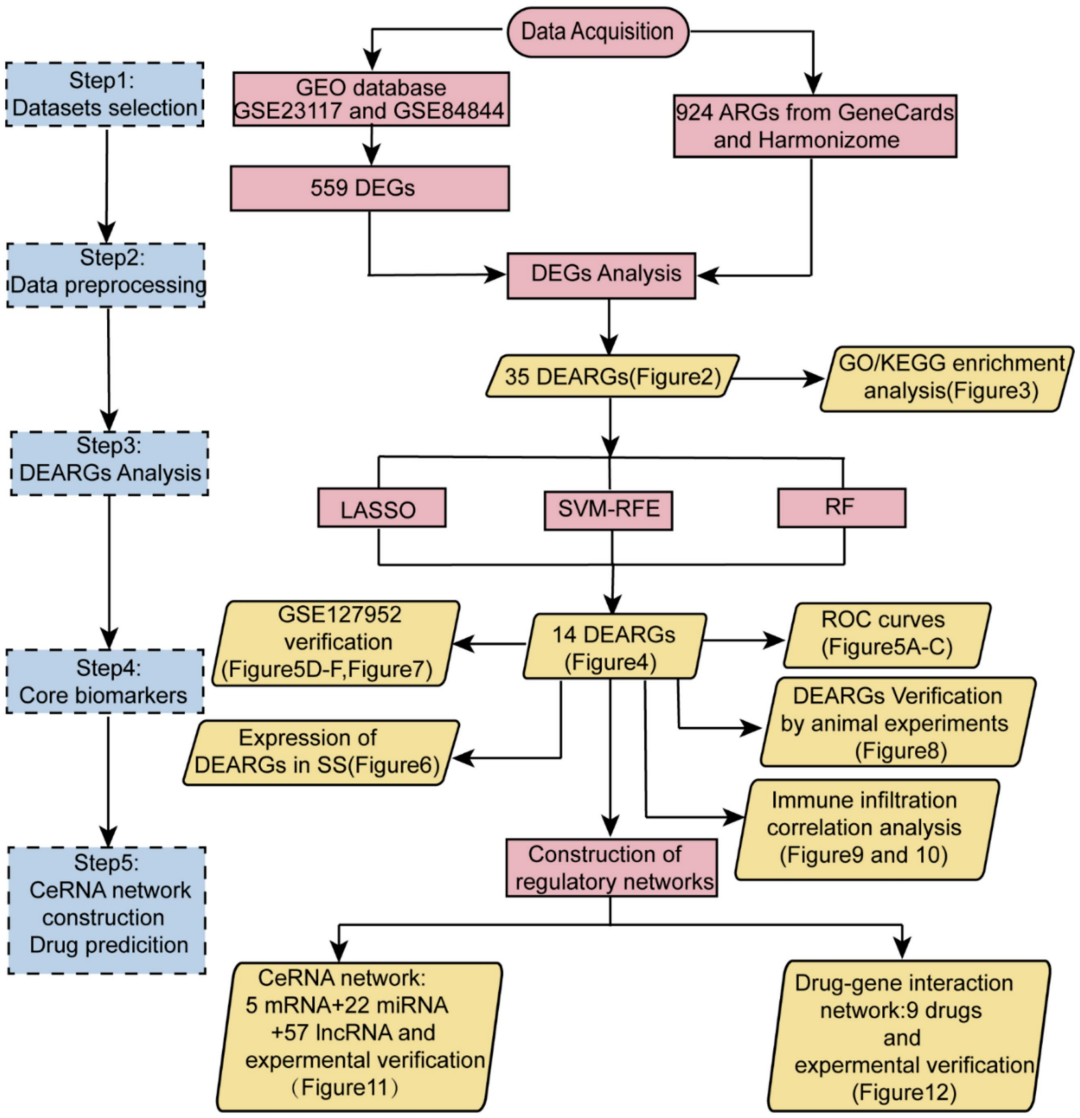
Detailed workflow of the study.

Expression data profiles for SS and healthy control samples were downloaded from the GEO database. The selection criteria were as follows: (1) all datasets were derived from the GPL570 [HG-U133_Plus_2] Affymetrix Human Genome U133 Plus 2.0 Array; (2) the species studied was *Homo sapiens*; (3) studies included SS patients and healthy controls; (4) studies contained at least 10 samples; and (5) samples originated from salivary glands or blood. The GSE23117 and GSE84844 datasets have been widely referenced and validated experimentally in numerous studies, supporting their reliability. Therefore, GSE23117 and GSE84844 were selected as training datasets, whereas GSE127952 served as the validation dataset. The GSE23117 dataset includes 15 samples (4 healthy controls and 11 SS cases), and GSE84844 includes 60 samples (30 healthy controls and 30 SS cases). ARGs were obtained from the GeneCards and Harmonizome databases. The detailed flow chart is shown in Figure 1.

### 2.2 Identification of differentially expressed genes

The “limma” package in R facilitated the analysis of DEGs in the GSE23117 and GSE84844 datasets. Significant differential expression criteria were set as log2|Fold Change (FC)| ≥ 1 and adj. *p*-value < 0.05. Subsequently, intersecting identified DEGs with ARGs revealed differentially expressed anoikis-related genes (DEARGs) in SS.

The limma package in R was employed to identify DEGs from the GSE23117 and GSE84844 datasets. The threshold for significant differential expression was defined as |log2 Fold Change (FC)| ≥ 1 and adjusted *p*-value< 0.05. Subsequently, the intersection between identified DEGs and ARGs yielded differentially expressed DEARGs associated with SS.

### 2.3 Enrichment analysis of DEARGs in GO and KEGG

Gene Ontology (GO) and Kyoto Encyclopedia of Genes and Genomes (KEGG) enrichment analyses were performed using the clusterProfiler package in R (9). GO enrichment analysis was conducted with the enrichGO function, utilizing genome-wide annotation provided by the Bioconductor annotation package (org.Hs.eg.db). KEGG pathway analysis was carried out using the enrichKEGG function (10, 11). Significant enrichment was defined as a *p*-value < 0.05.

To control false-positive risks arising from multiple comparisons in GO and KEGG enrichment analyses, the Benjamini–Hochberg method was applied to correct original *p*-values for multiple hypothesis testing, with an adjusted q-value (FDR) threshold of <0.05 used to define statistical significance.

### 2.4 Feature gene selection by machine learning

Three machine learning algorithms were applied to screen for key ARGs associated with SS. Least absolute shrinkage and selection operator (LASSO) logistic regression, implemented using the glmnet package in R, identified the optimal penalty parameter to minimize binomial deviation (12). Support vector machine-recursive feature elimination (SVM-RFE), conducted using the R packages “e1071,” “kernlab,” and “caret,” recursively eliminated features and calculated weights (13). Random forest (RF), an ensemble learning method executed with the randomForest package in R, identified the top ten genes ranked by importance scores (14). Genes consistently identified by multiple machine learning methods were selected as the final ARGs.

To ensure the robustness and generalizability of feature selection, this study combined LASSO, SVM-RFE, and Random Forest methods. LASSO employs L1 regularization for feature sparsity and effectively eliminates redundant features; SVM-RFE iteratively removes less informative variables, emphasizing direct impact on classification performance; Random Forest, leveraging an ensemble learning framework, evaluates the global contribution of variables through feature importance ranking. These methods complement each other regarding sparsity, nonlinear adaptability, and stability, thereby enhancing the scientific rigor and robustness of feature selection. To further mitigate chance bias, features selected by≥2 methods were considered stably important and included in subsequent modeling analyses. This threshold was chosen to balance statistical rigor and clinical interpretability.

### 2.5 ROC curve analysis of feature genes

The diagnostic performance of selected feature genes for SS was assessed by receiver operating characteristic (ROC) curve analysis using the pROC and ggplot2 packages in R. The area under the curve (AUC) values, ranging from 0.5 to 1, reflect diagnostic accuracy, with higher values indicating greater predictive power (15). Dataset GSE127952 served as the external validation set to evaluate the diagnostic efficacy of these candidate genes.

### 2.6 Validation of feature gene expression

The expression levels of feature genes in disease and control conditions were validated using the SS-related dataset (GSE127592). Analysis and visualization of gene expression data were conducted using the ggplot2 package in R.

### 2.7 Immune cell infiltration and differential analysis

Immune cell infiltration was evaluated in the selected samples using the CIBERSORT algorithm. The proportions of 22 immune cell subtypes in SS and control samples were compared (16), and subtypes with a CIBERSORT *p*-value < 0.05 were selected for further analysis. Differences in immune cell proportions between SS and control groups were assessed using the Wilcoxon test (*p* < 0.05 as statistically significant). Results were visualized as heatmaps generated by the ggplot2 package. Correlations among the 22 infiltrating immune cell types were visualized using the corrplot package. In addition, associations between immune cell proportions and feature gene expression were analyzed and visualized using the ggplot2 (version 3.3.5), ggpubr, and ggExtra (version 0.9) packages in R, considering *p* < 0.05 statistically significant.

### 2.8 Regulatory mechanisms of potential feature genes

Candidate miRNAs targeting feature genes were predicted using the miRanda, miRDB, and TargetScan databases. The starBase database was used to identify lncRNAs associated with these miRNAs. Subsequently, a competing endogenous RNA (ceRNA) network comprising candidate miRNAs and lncRNAs was constructed visualized using Cytoscape software.

### 2.9 Animals and experimental design

A spontaneous SS mouse model (NOD/Ltj mice) was employed in the study. Female specific pathogen-free (SPF) NOD mice and BALB/c mice (8 weeks old, weighing 18–23 g) were purchased from Beijing Huafukang Biotechnology Co., Ltd. and Beijing Weitongxing Biotechnology Co., Ltd. (Beijing, China), respectively. Animals were housed under controlled conditions (25 ± 2 °C) with free access to standard food and water. The experimental protocol was approved by the Animal Ethics Committee of Shandong University of Traditional Chinese Medicine. NOD/Ltj mice represented the SS group, whereas BALB/c mice were used as controls. Average water intake (mg/mL) and salivary flow (μg/g) were recorded at weeks 8, 10, 12, 14, and 16. At 16 weeks, mice were fasted overnight, anesthetized, and euthanized via cervical dislocation following orbital blood collection. The submandibular glands were harvested for subsequent histological evaluation and experimental analyses. All animal procedures adhered strictly to the Guide for the Care and Use of Laboratory Animals (National Institutes of Health Publication No. 85–23, revised 1996).

### 2.10 Histology and immunohistochemistry

Collected submandibular glands were fixed in 4% paraformaldehyde for at least 24 h, embedded in OCT medium, and sectioned (8–10 μm thickness). Sections were stained with hematoxylin and eosin (H&E) according to the manufacturer’s guidelines. Masson’s trichrome staining was performed to the assess collagen deposition. Slides were examined and imaged using fluorescence microscopy, and images were quantitatively analyzed Image J software (version 1.8.0).

### 2.11 Caspase-3 activity assay

Caspase-3 activity in the mandibular gland tissues was measured using a Caspase-3 Assay Kit (Cat No. ab39401, Abcam) according to the manufacturer’s instructions. Briefly, tissues were lysed with lysis buffer on ice (10 min). Protein concentrations were determined and adjusted to 1 μg/μL. Reaction mixtures, containing 50 μL sample, 50 μL reaction buffer (with 10 mM DTT), and 5 μL 4 mM DEVD-pNA substrate, were incubated at 37 °C for 120 min, and absorbance was measured at 405 nm using a microplate reader.

### 2.12 Quantitative real-time PCR (qRT-PCR)

Total RNA was isolated from submandibular gland tissues using Trizol reagent (R401-01, Vazyme) and reverse-transcribed to complementary DNA (cDNA) using a reverse transcription kit. qRT-PCR was performed using SYBR Green reagent on a real-time PCR system. Target mRNA expression levels were normalized to the internal control (*β*-actin). Primer sequences for qRT-PCR are provided in Table 1.

**Table 1:** Primer sequences for PCR.

RNA	Forward	Reverse
*Mapk3*	AGGCTTCTCCCACTCCAATCCC	TCCATTCCAGAACGGTCTACCAGAG
*Il15*	AAGGAATGTGAGGAGCTGGAGGAG	TGCAGTCAGGACGTGTTGATGAAC
*S100a9*	TGGACACAAACCAGGACAATCAGC	TTCCCACAGCCTTTGCCATGAC
*Ifi27*	TGGACTCTCCGTGCCATCTACTG	TCGCCATATCTGCCACCTCTGTC
*Cxcl10*	TCGCTCAAGTGGCTGGGATGG	GGGAGGACAAGGAGGGTGTGG
*β-actin*	CCTTCCGTGTTCCTACCCC	GCCCAAGATGCCCTTCAGT

### 2.13 Western blot (WB) analysis

Total protein from mouse submandibular glands was extracted using a Minute™ Total Protein Extraction Kit for Bone Tissue (Invent, USA) following the manufacturer’s instructions. Protein lysates were prepared with RIPA buffer containing PMSF, protease inhibitor cocktail, and phosphatase inhibitors (Epizyme, China). Protein concentration was measured using a BCA assay kit (Beyotime, China). Samples (40 μg protein) were separated by SDS-PAGE (Epizyme, China) and transferred onto PVDF membranes (0.22/0.45 μm, Millipore, USA). Membranes were blocked with 5% skim milk at room temperature (2 h) and incubated at 4 °C for 16–18 h with primary antibodies against Phospho-MAPK3 (Cell Signaling Technology Cat#4370) and MAPK3 (Cell Signaling Technology Cat#4695). Membranes were then incubated with HRP-conjugated secondary antibodies at room temperature (1 h), and antibody–antigen complexes were visualized using an ECL kit (Millipore, USA) and a multicolor fluorescence imaging system (Amersham Imager 600, GE, USA). After stripping (Epizyme, China), membranes were re-blocked (5% skim milk, 1 h) and incubated with a *β*-actin antibody (Proteintech, China) at 4 °C for 4 h. Protein bands were quantified with Image J software.

### 2.14 Enzyme-linked immuno sorbent assay (ELISA)

Serum levels of IL15, S100A9, IFI27, and CXCL10 were measured using corresponding ELISA kits (Elabscience, China) following the manufacturer’s protocols.

### 2.15 Statistical analysis

Data were presented as mean ± standard deviation (SD). Statistical analyses were performed using SPSS Statistics 25 (IBM, Chicago, IL, USA) and GraphPad Prism 8.0 (GraphPad Software, USA). Comparisons between groups were conducted by one-way ANOVA, and *p*-values < 0.05 indicated statistical significance. All experiments were conducted at least three times.

## 3 Results

### 3.1 Differential expression analysis and identification of ARGs in SS

**Figure 2:**
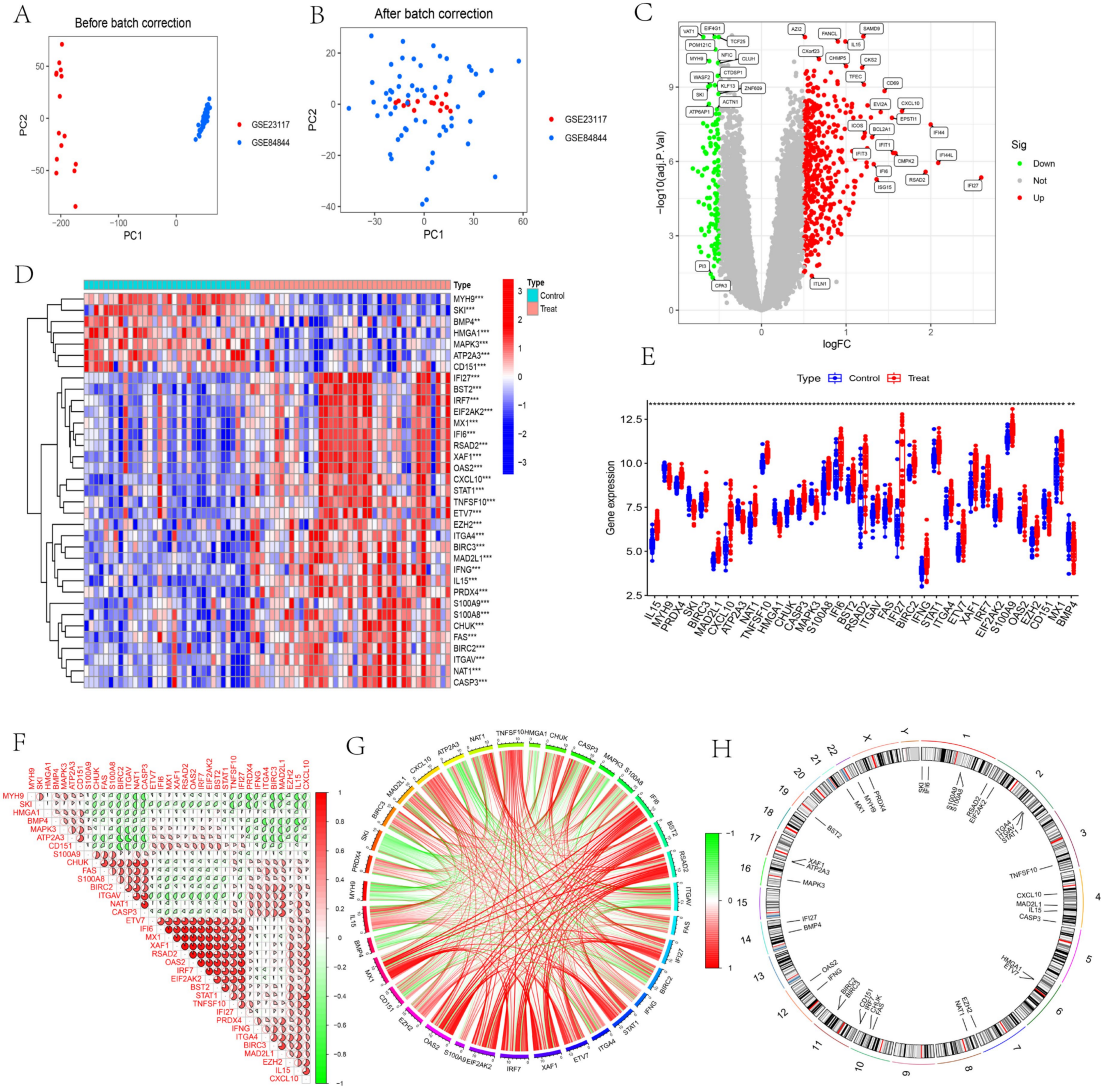
Expression spectrum of ARGs in SS. **(A)** PCA before batch correction; **(B)** PCA after batch correction; **(C)** Volcano plot of DEARGs; **(D)** Heatmap illustrating gene expression; **(E)** Box plot of expression differences; **(F)** Correlation heatmap; **(G)** Circular correlation plot; **(H)** Chromosomal locations of DEARGs.

Distinct clustering patterns were observed between the GSE23117 and GSE84844 datasets prior to batch correction, however, these patterns converged significantly after correction (Figures 2A,B). As shown in Figures 2A,B, samples from different batches showed distinct clustering along the first two principal components prior to batch correction, indicating a pronounced batch effect. After correction using the ComBat method, the distribution of samples across batches became more uniform, and clustering was notably diminished. Further quantitative analysis revealed that variance explained by batch factors in the first two principal components decreased markedly from 28.6 to 5.2%, significantly reducing the batch contribution to overall variance. Additionally, the *F*-value for inter-batch differences decreased from 15.4 to 2.1, indicating effective removal of batch effects and improved comparability across samples. Using the limma package, 559 DEGs were identified in the SS datasets, including 434 upregulated and 125 downregulated genes (Figure 2C). Among the 924 ARGs obtained from the GeneCards and Harmonizome databases, 35 DEARGs were obtained through intersection with the identified DEGs (Figures 2D,E). Chromosomal locations of these 35 DEARGs were visualized (Figure 2H). Additionally, correlation analysis revealed interactions among DEARGs, notably identifying significant antagonistic interactions between *MX1* and genes such as *RSAD2, OAS2, IRF7, EIF2AK2*, and *BST2*. Conversely, *MYH9* exhibited a strong synergistic relationship with *NAT1, CASP3, PRDX4, BIRC3,* and *MAD2L1* (Figures 2F,G).

### 3.2 GO and KEGG pathway enrichment analysis of DEARGs

**Figure 3:**
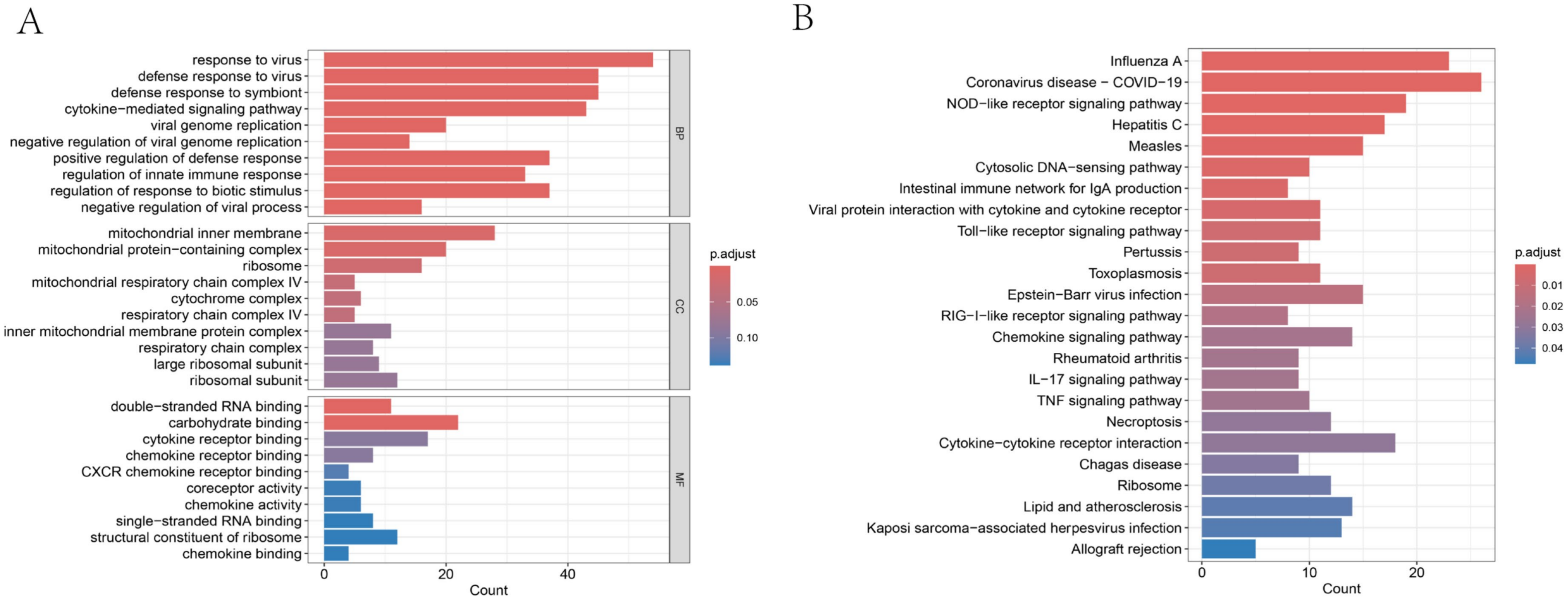
Enrichment analyses of DEARGs. **(A)** Top 10 enriched GO terms in BP, CC, and MF; **(B)** KEGG pathway enrichment analysis.

GO and KEGG enrichment analyses were conducted to explore the biological functions associated with SS-related DEARGs. The GO analysis revealed significant enrichment of DEARGs in biological processes (BP), molecular functions (MF), and cellular components (CC), primarily involving immune-related activities such as regulation of innate immune response, cytokine-mediated signaling pathways, mitochondrial inner membrane, inner mitochondrial membrane protein complexes, cytokine receptor binding, and chemokine receptor binding (Figure 3A). These findings suggest a potential link between SS pathogenesis and mitochondrial membrane function, cytokine receptor activity, and pathways mediated by innate immune responses signaling pathways. KEGG analysis indicated enrichment in pathways, including the NOD-like receptor (NLR) signaling pathway, coronavirus disease (COVID-19), intestinal immune network for IgA production, Toll-like receptor (TLR) signaling pathway, chemokine signaling pathway, rheumatoid arthritis, IL-17 signaling pathway, TNF signaling pathway, and necroptosis (Figure 3B). These results highlight the association between SS development and inflammation, viral infections, and necroptotic signaling pathways.

### 3.3 Feature gene selection using machine learning

**Figure 4:**
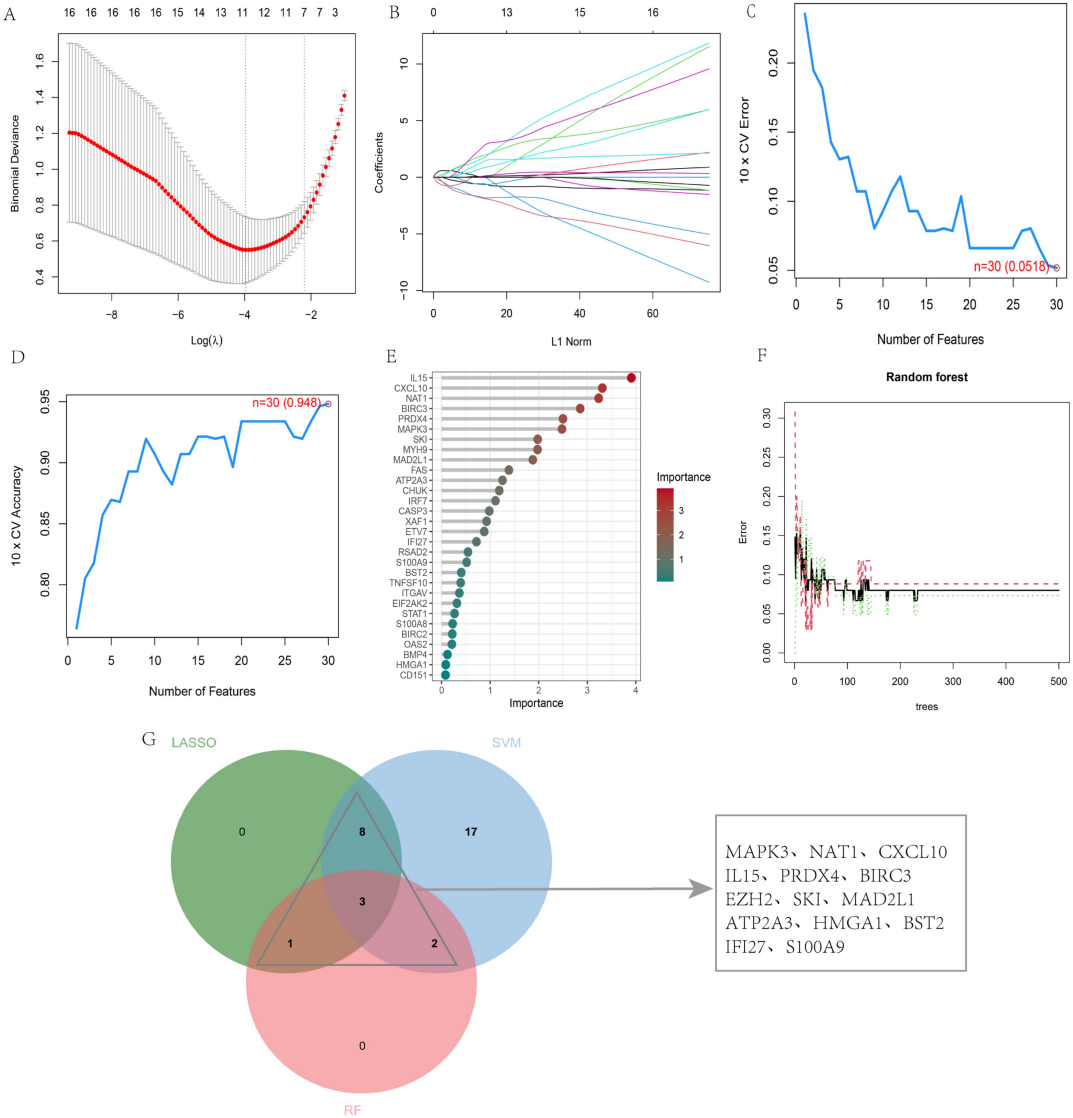
Feature gene selection for SS via machine learning algorithms. **(A)** LASSO regression plot; **(B)** Cross-validation for LASSO; **(C,D)** SVM-RFE feature selection plots; **(E)** Gene importance ranking using RF; **(F)** Relationship between RF trees and error rate; **(G)** Venn diagram of intersecting feature genes.

Feature genes related to SS were identified among DEARGs using LASSO regression, SVM-RFE, and RF algorithms. LASSO regression identified 12 candidate genes from the 35 DEARGs, significantly facilitating SS diagnosis (Figures 4A,B). SVM-RFE analysis yielded 30 candidate feature genes (Figures 4C,D). The RF algorithm ranked the 35 DEARGs according to variable importance, focusing on genes with a MeanDecreaseGini > 2 (Figures 4E,F). Finally, 14 overlapping genes were identified by intersecting the outcomes of these three algorithms (Figure 4G).

### 3.4 Validation of feature genes

**Figure 5:**
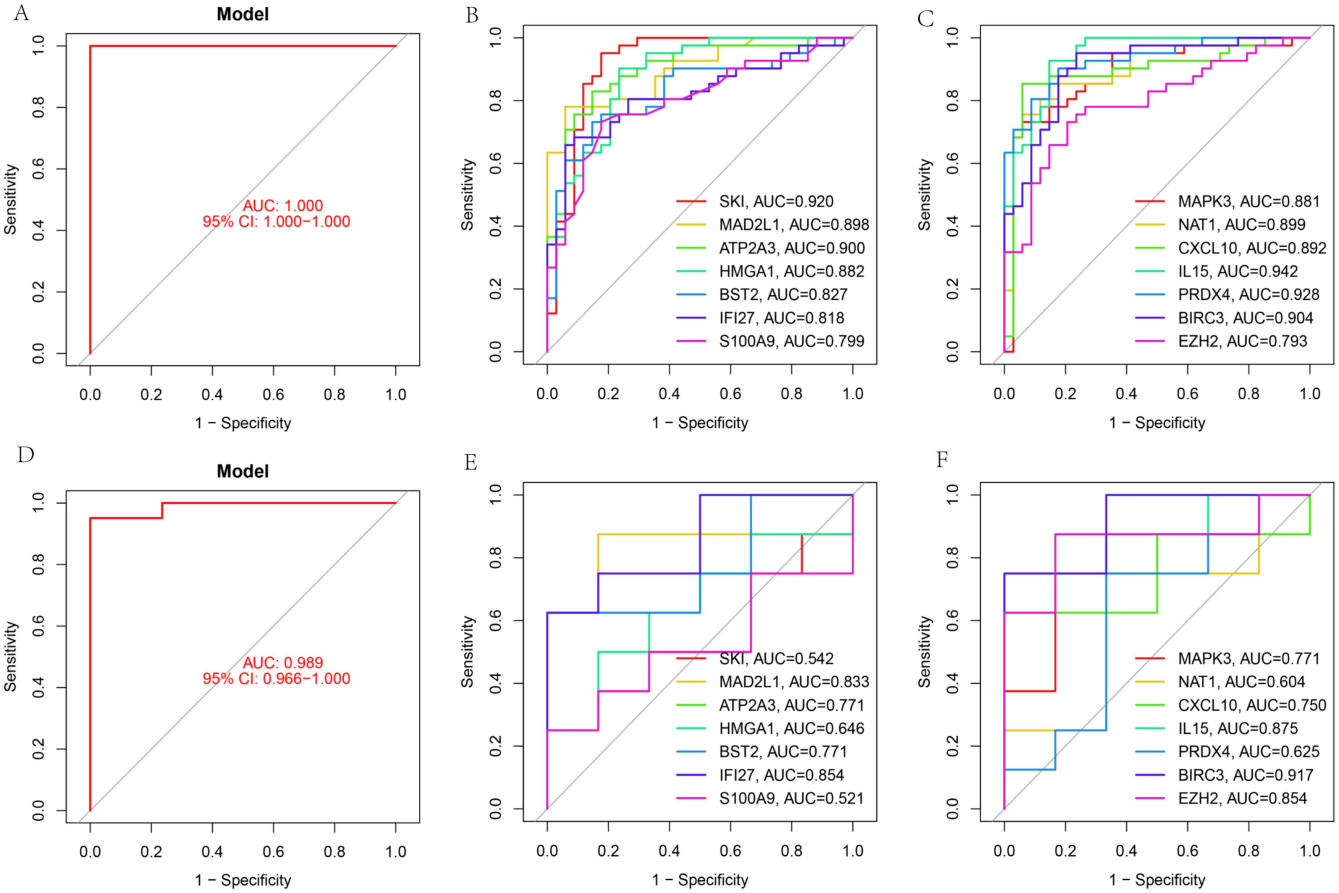
Validation of feature genes. **(A)** ROC curves for diagnostic models in combined datasets (GSE23117 and GSE84844); **(B,C)** Individual ROC curves; **(D)** ROC curves for diagnostic models in GSE127952; **(E,F)** ROC curves in validation dataset.

ROC curve analysis was performed to assess the diagnostic utility of *MAPK3, NAT1, CXCL10, IL15, PRDX4, BIRC3, EZH2, SKI, MAD2L1, ATP2A3, HMGA1, BST2, IFI27,* and *S100A9* using the combined GSE23117 and GSE84844 datasets. All evaluated genes exhibited AUC values greater than 0.7 in the combined dataset, demonstrating their strong diagnostic potential for SS (Figures 5A–C). Further validation using dataset GSE127952 revealed that, except for *SKI* and *S100A9*, the remaining genes displayed AUC values greater than 0.6 (Figures 5D–F), reinforcing the reliability of these findings.

Although model performance evaluation indicated ROC-AUC values above 0.7 for all features in the training set, suggesting robust internal discriminative ability, the reduced AUC values (0.6–0.7) observed for certain gene markers in the independent validation set suggest potential overfitting. This phenomenon is common in high-dimensional biomedical research with limited sample sizes, possibly arising from insufficient training samples, feature selection influenced by current data distributions, and clinical or biological heterogeneity within validation cohorts. Although validation set AUC values approaching 0.7 retain some discriminative capability, their clinical translation value requires further verification. Future studies will improve model robustness and generalizability by increasing sample sizes, incorporating multicenter validation cohorts, and integrating multi-omics data, thereby facilitating clinical diagnostics and risk stratification.

**Figure 6:**
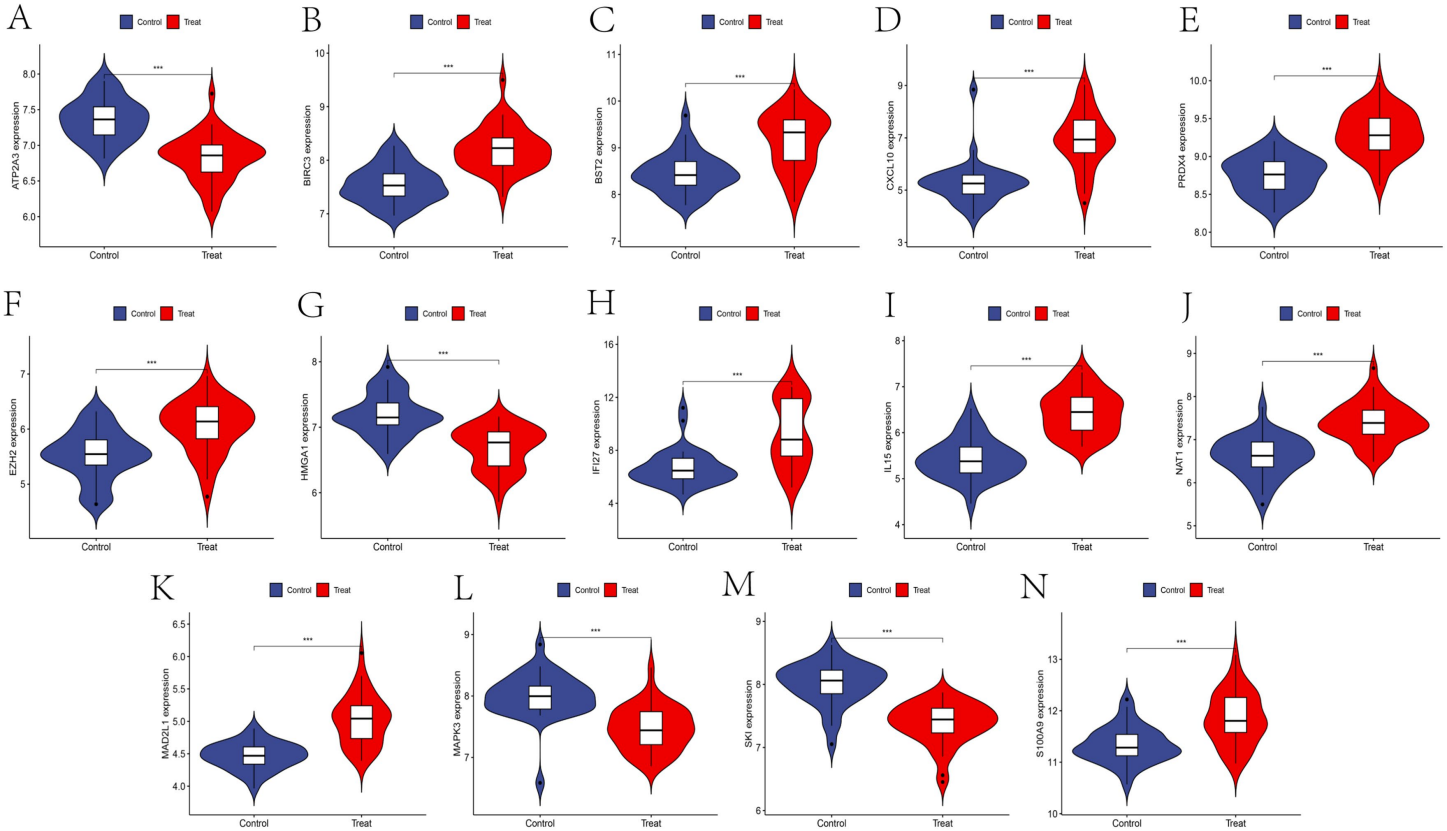
**(A–N)** Expression of feature genes in datasets GSE23117 and GSE84844.

**Figure 7:**
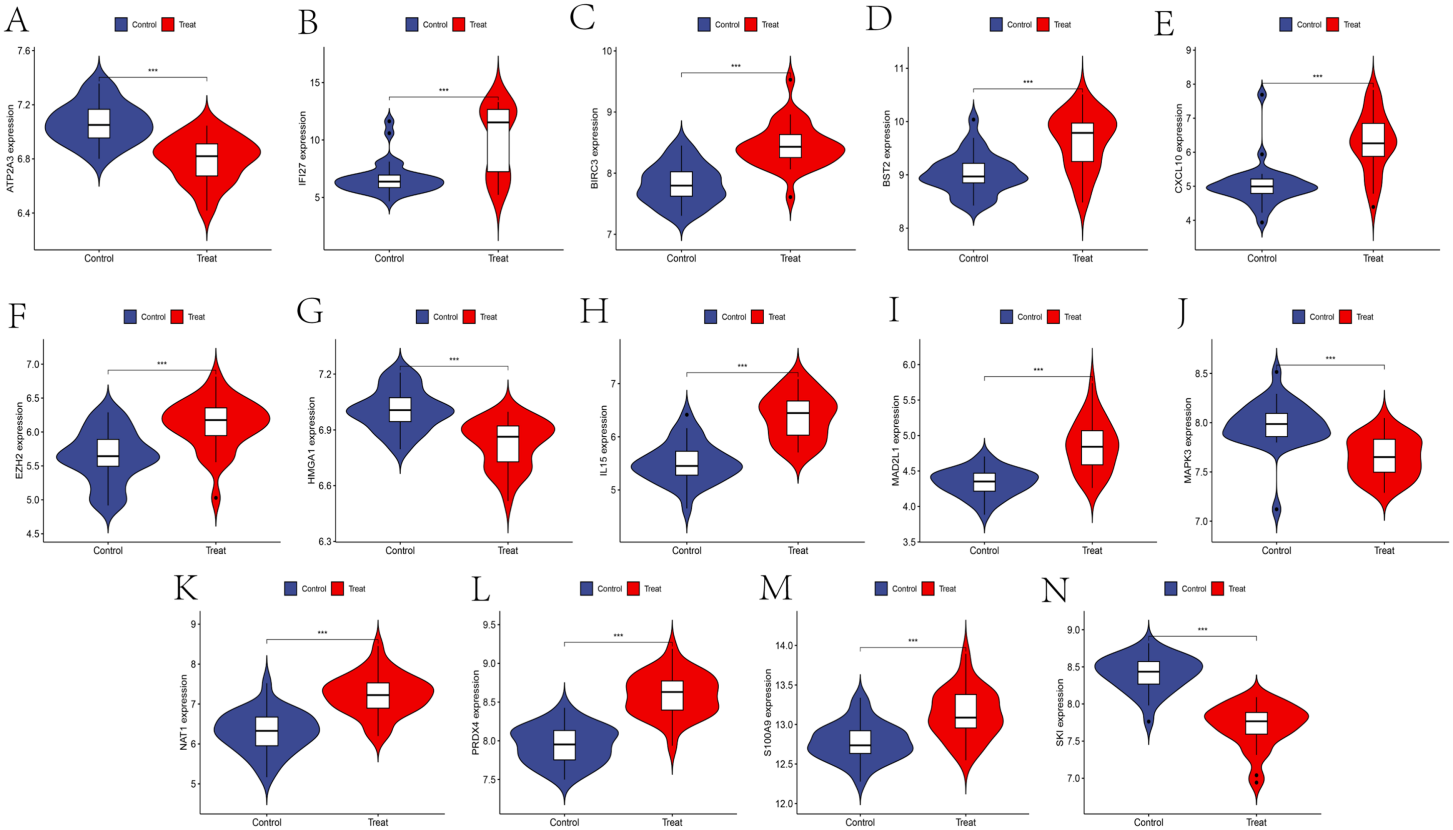
**(A–N)** Expression of feature genes in validation dataset GSE127952.

To confirm the expression patterns of these 14 feature genes in SS, further expression analysis was conducted using the SS-related training and validation datasets. Except for *ATP2A3, HMGA1, MAPK3,* and *SKI*, the remaining feature genes were predominantly upregulated in SS patients (Figure 6). Consistent expression trends were also validated in the external dataset (Figure 7), providing additional confirmation of our conclusions.

### 3.5 *In vivo* validation of feature gene expression in SS mouse

**Figure 8:**
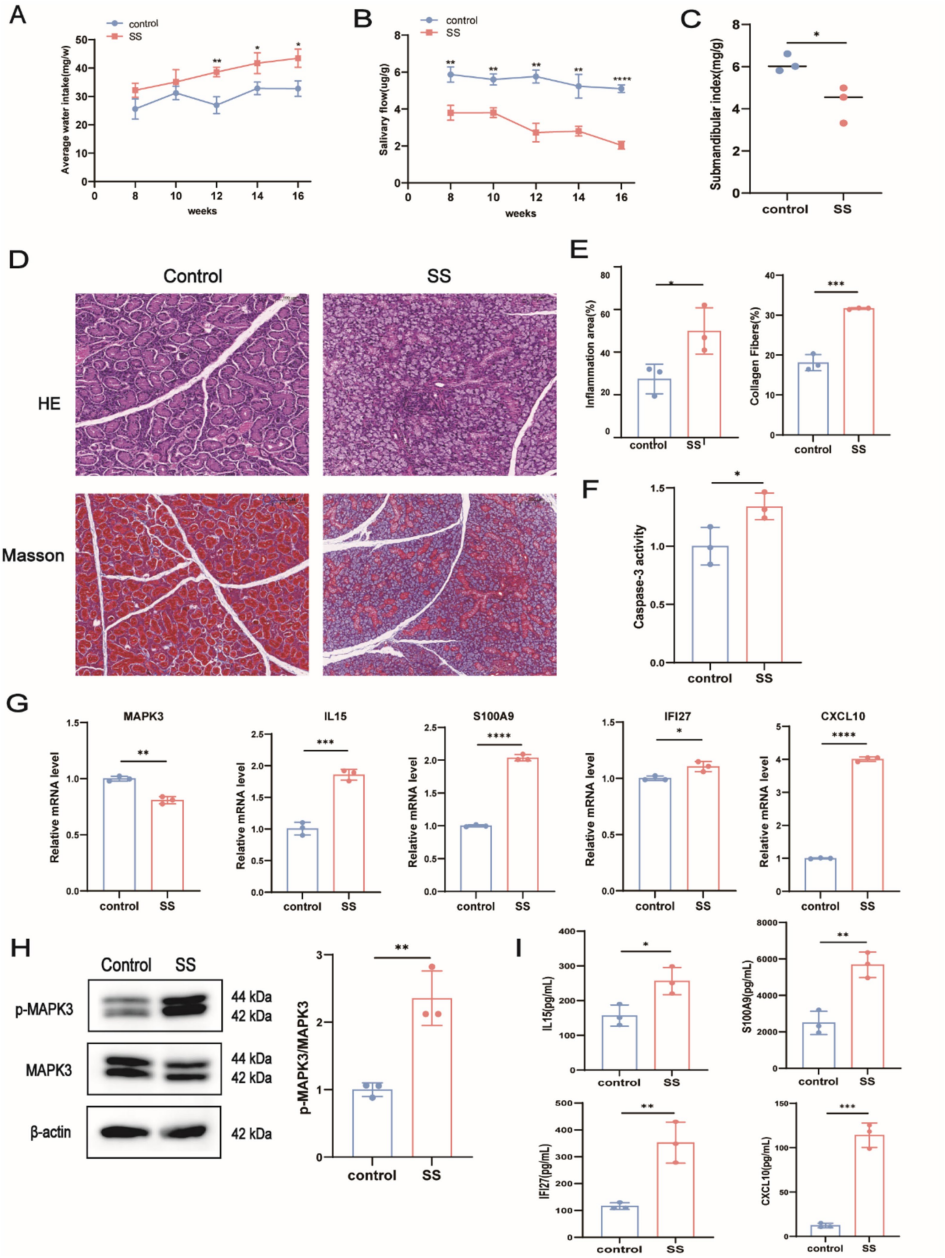
Validation of feature gene expression through *in vivo* experiments. **(A)** Average water intake of mice from 8 to 16 weeks; **(B)** Salivary flow rate of mice from 8 to 16 weeks; **(C)** Submandibular gland index at week 16; **(D)** Representative images of HE and Masson staining in submandibular glands; **(E)** Quantitative analysis of HE and Masson staining; **(F)** Caspase-3 activity assay; **(G)** RT-qPCR analysis of *Mapk3, Il15, S100a9, Ifl27,* and Cxcl10 expression; **(H)** WB analysis of p-MAPK3 expression; **(I)** ELISA quantification of IL15, S100A9, IFI27, and CXCL10 levels in serum. ^ns^*p* > 0.05, *p < 0.05, **p < 0.01, ***p < 0.001, ****p < 0.0001.

Previously, 14 feature genes were identified. Database searches indicated that *NAT1, BIRC3, EZH2, MAD2L1, ATP2A3, HMGA1,* and *BST2* are cancer-associated genes. Although *SKI* and *PRDX4* have been linked to various inflammatory diseases, their roles in SS remain unclear and were therefore excluded from further analysis. Consequently, the study focused on *MAPK3, IL15, S100A9, IFI27,* and *CXCL10*. To validate their expression, an SS mouse model was established. As shown in Figures 8A,B, water intake gradually increased (*p* < 0.05), whereas salivary flow rate gradually decreased (*p* < 0.05) in the SS group compared to the control group. Additionally, at 16 weeks, the submandibular gland index was significantly reduced in SS mice (*p* < 0.05, Figure 8C). HE and Masson staining revealed markedly increased inflammation in submandibular glands from SS mice (*p* < 0.05, Figures 8D–F), confirming successful establishment of the mouse model. Furthermore, caspase-3 activity was significantly elevated in the submandibular glands of SS mice (Figure 8F). Next, total protein, and serum samples were extracted from the submandibular glands. qPCR revealed significant upregulation of *S100a9, Ifi27, Cxcl10,* and *Il15* (*p* < 0.05), whereas *Mapk3* expression was significantly decreased (*p* < 0.05) in SS mice (Figure 8G). WB analysis showed increased phosphorylation of MAPK3 (Figure 8H), and ELISA confirmed significantly elevated serum levels of S100A9, IFI27, CXCL10, and IL15 in SS mice (Figure 8I). These findings further validate our bioinformatics analyses.

### 3.6 Immune cell infiltration analysis in SS

**Figure 9:**
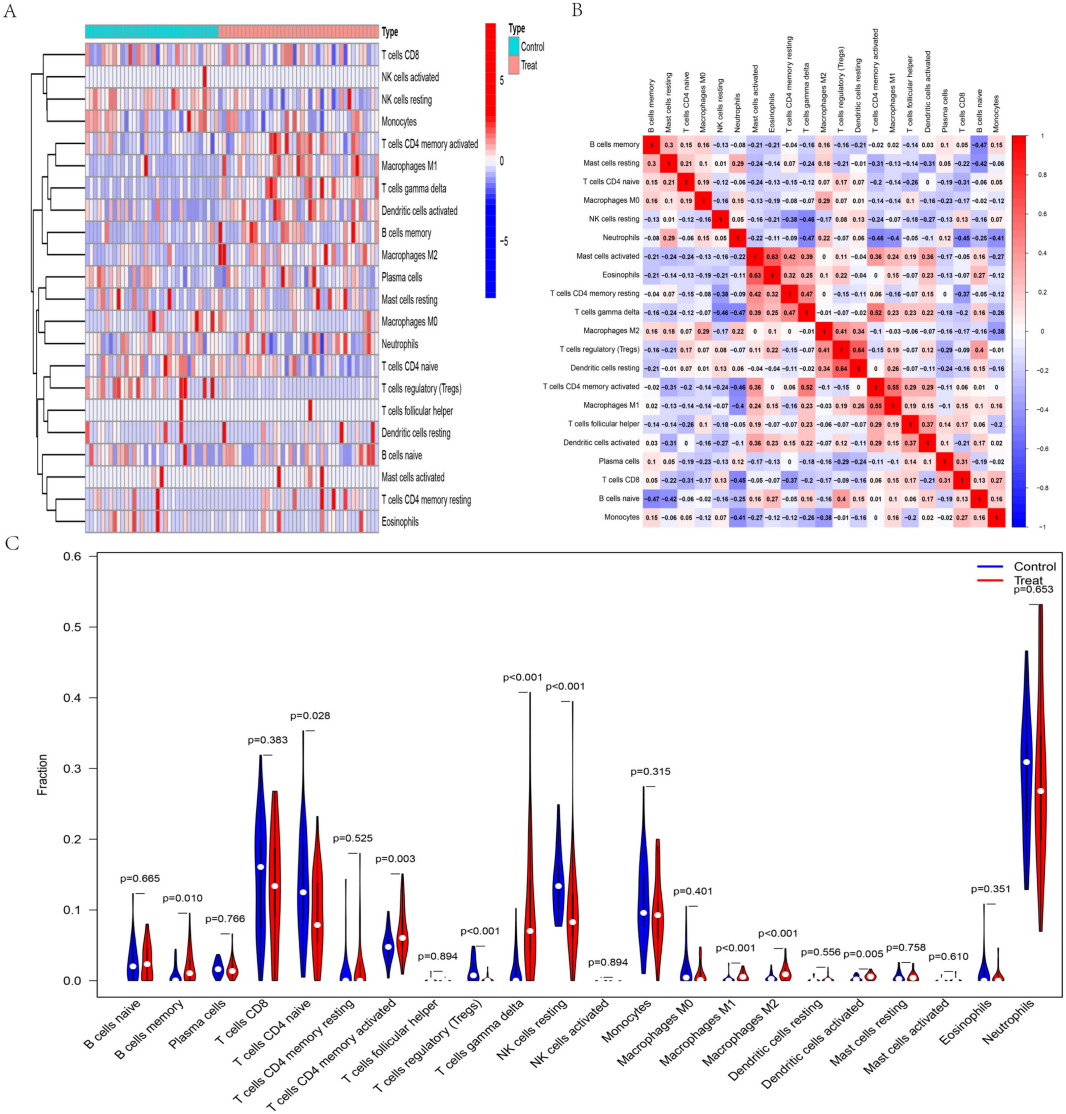
Immune cell infiltration analysis. **(A)** Correlation heatmap of 22 infiltrating immune cells; **(B)** Heatmap illustrating immune cell correlations; **(C)** Violin plots comparing the proportions of 22 immune cell types.

Considering the f strong association between DEARGs and immune-inflammatory pathways in SS, the CIBERSORT algorithm was utilized used to assess immune cell infiltration in SS patients. A correlation heatmap for immune cells was generated (Figure 9A), displaying positive (red) and negative (blue) correlations, with color intensity representing correlation strength. Analysis of correlations among 22 immune cell types identified a significant negative correlation between resting dendritic cells and naïve B cells (*r* = −0.47, *p* < 0.05) (Figure 9B). Additionally, memory B cells, naïve CD4 + T cells, activated memory CD4 + T cells, regulatory T cells, gamma-delta T cells, resting NK cells, M1 macrophages, M2 macrophages, and activated dendritic cells differed significantly between SS patients and healthy controls (Figure 9C). In this study, the CIBERSORT method was employed to systematically analyze immune cell infiltration within salivary gland tissues. Although CIBERSORT provides clear insights into the immune microenvironment, inherent limitations must be acknowledged. Firstly, CIBERSORT utilizes the LM22 signature matrix, derived from peripheral blood immune cells, which may not fully represent the immune cell composition unique to salivary gland tissues, potentially affecting the accuracy of the inferred results. Secondly, salivary glands are highly heterogeneous exocrine organs, comprising epithelial cells, acinar cells, and various immune cell types, which may complicate deconvolution analyses. Additionally, CIBERSORT provides relative rather than absolute cell proportions, necessitating caution during interpretation. Therefore, our conclusions require further validation through subsequent experiments and multi-omics approaches.

### 3.7 Correlation between feature genes and immune cell infiltration

**Figure 10:**
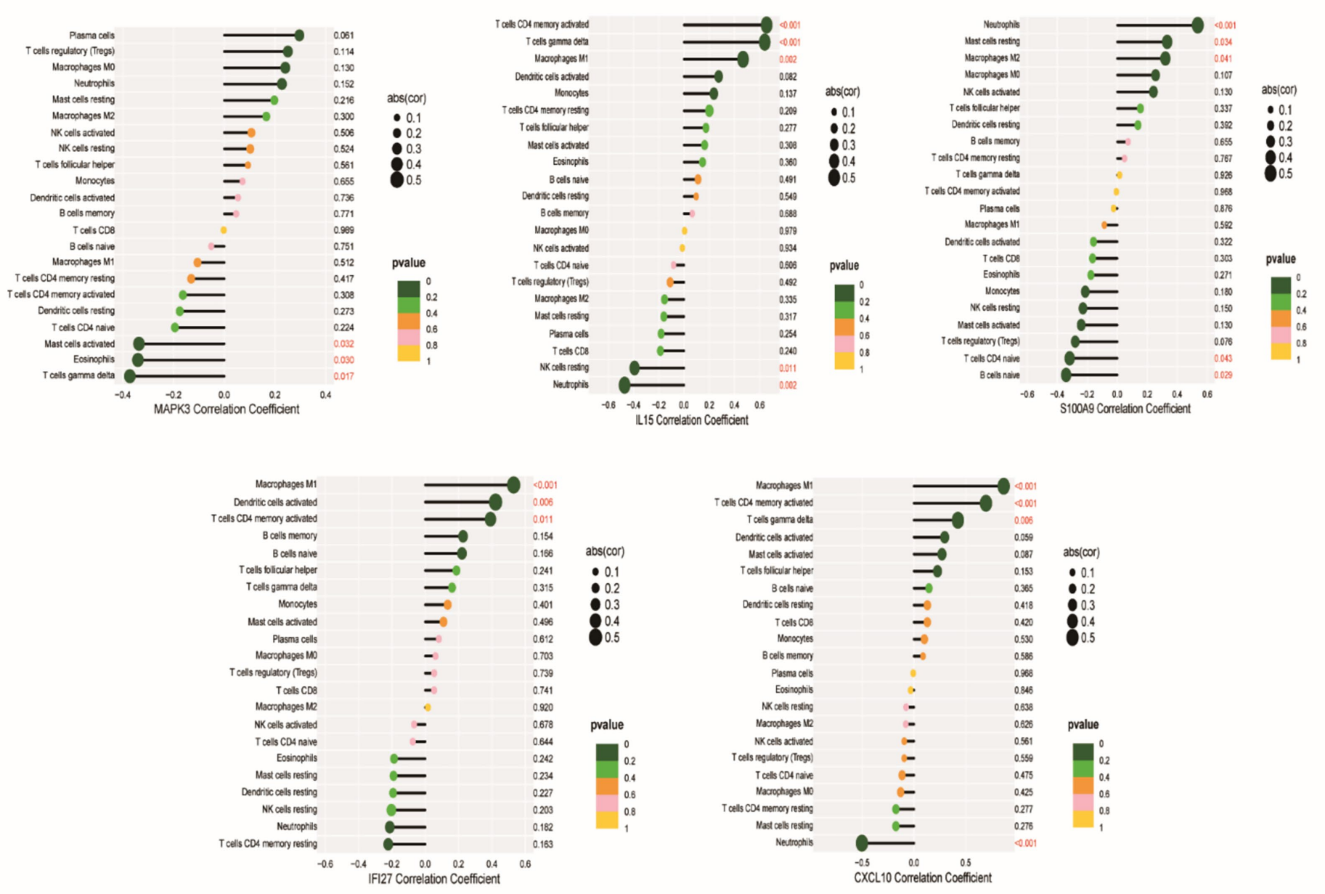
Correlation analysis between feature genes and immune cell infiltration.

To further explore the relationship between feature genes and immune cell infiltration, subsequent analyses revealed that *CXCL10, IFI27,* and *IL15* were positively correlated with M1 macrophage abundance. *CXCL10* and *IL15* showed negative correlations with neutrophils, while *S100A9* demonstrated a positive correlation. *CXCL10, IFI27,* and *IL15* positively correlated with activated memory CD4 + T cell abundance. Additionally, *CXCL10, IL15,* and *MAPK3* positively correlated with gamma-delta T cells (Figure 10). Overall, these correlations suggest that the expression of feature genes may be linked to immune cell infiltration in SS, potentially implicating them in the immunological mechanisms underlying SS pathogenesis. However, these findings reflect associative trends between gene expression and immune cell abundance, and causal relationships and underlying molecular mechanisms require further functional validation.

### 3.8 Regulatory network analysis of feature genes

**Figure 11:**
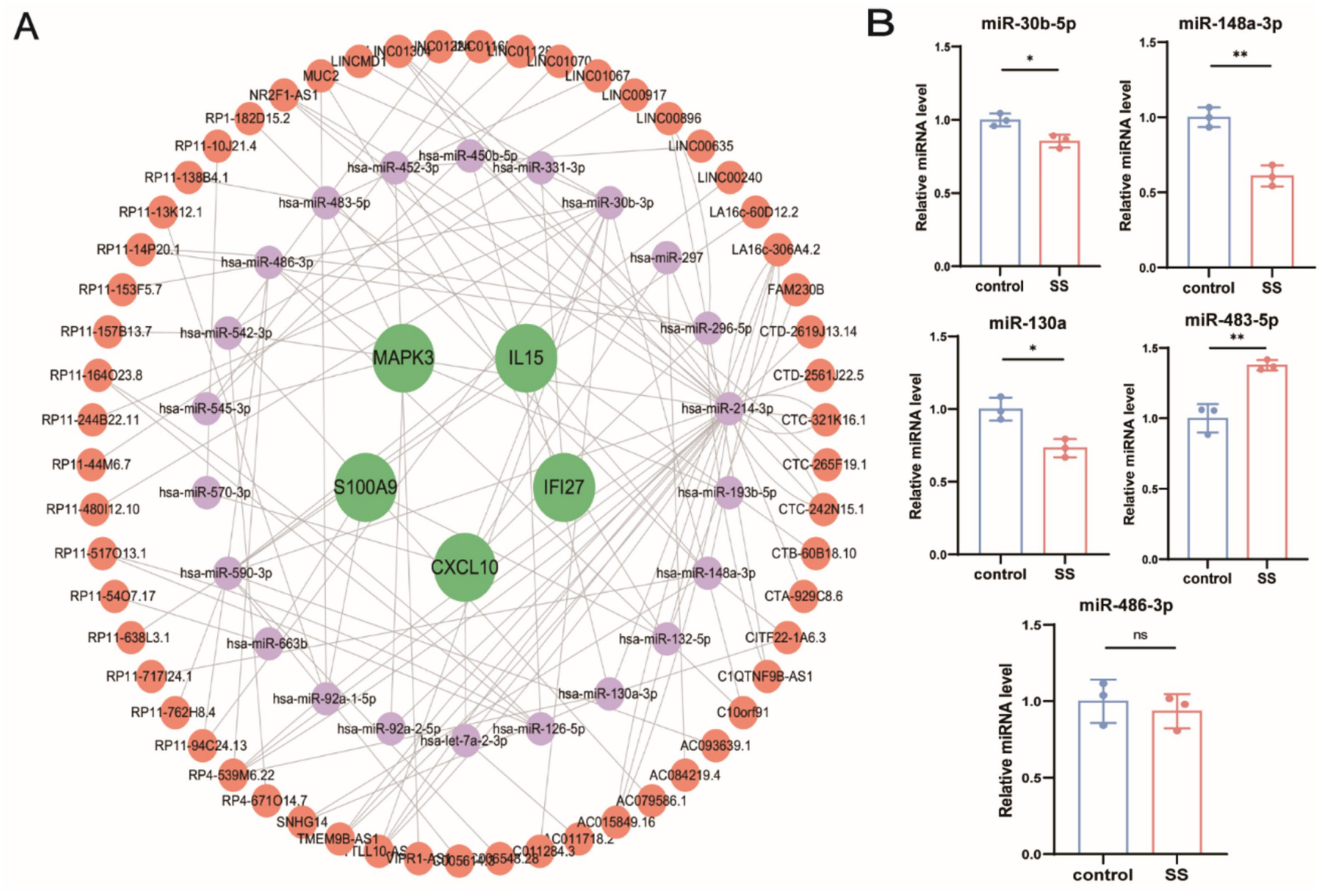
**(A)** Construction of the ceRNA network for feature genes. Green circles represent mRNAs, purple circles represent miRNAs, and brown circles represent lncRNAs. **(B)** RT-qPCR validation of miRNA expression. ^ns^*p* > 0.05, *p < 0.05, **p < 0.01, ***p < 0.001, ****p < 0.0001.

In the ceRNA model, lncRNAs and mRNAs that share miRNA binding sites function as competing endogenous RNAs, modulating shared miRNAs (17). MiRNAs binding to feature genes were predicted using MiRanda, miRDB, and TargetScan databases. Remarkably, miRNAs corresponding to only five feature genes were predicted. Using the Starbase database, 22 miRNAs regulating 57 lncRNAs were identified. Subsequently, a ceRNA network comprising 5 mRNAs, 22 miRNAs, and 57 lncRNAs was constructed (Figure 11A). Additionally, five SS-related miRNAs were selected for validation (Figure 11B) to explore their potential as biomarkers for SS.

### 3.9 Identification of candidate drugs targeting diagnostic genes

**Figure 12:**
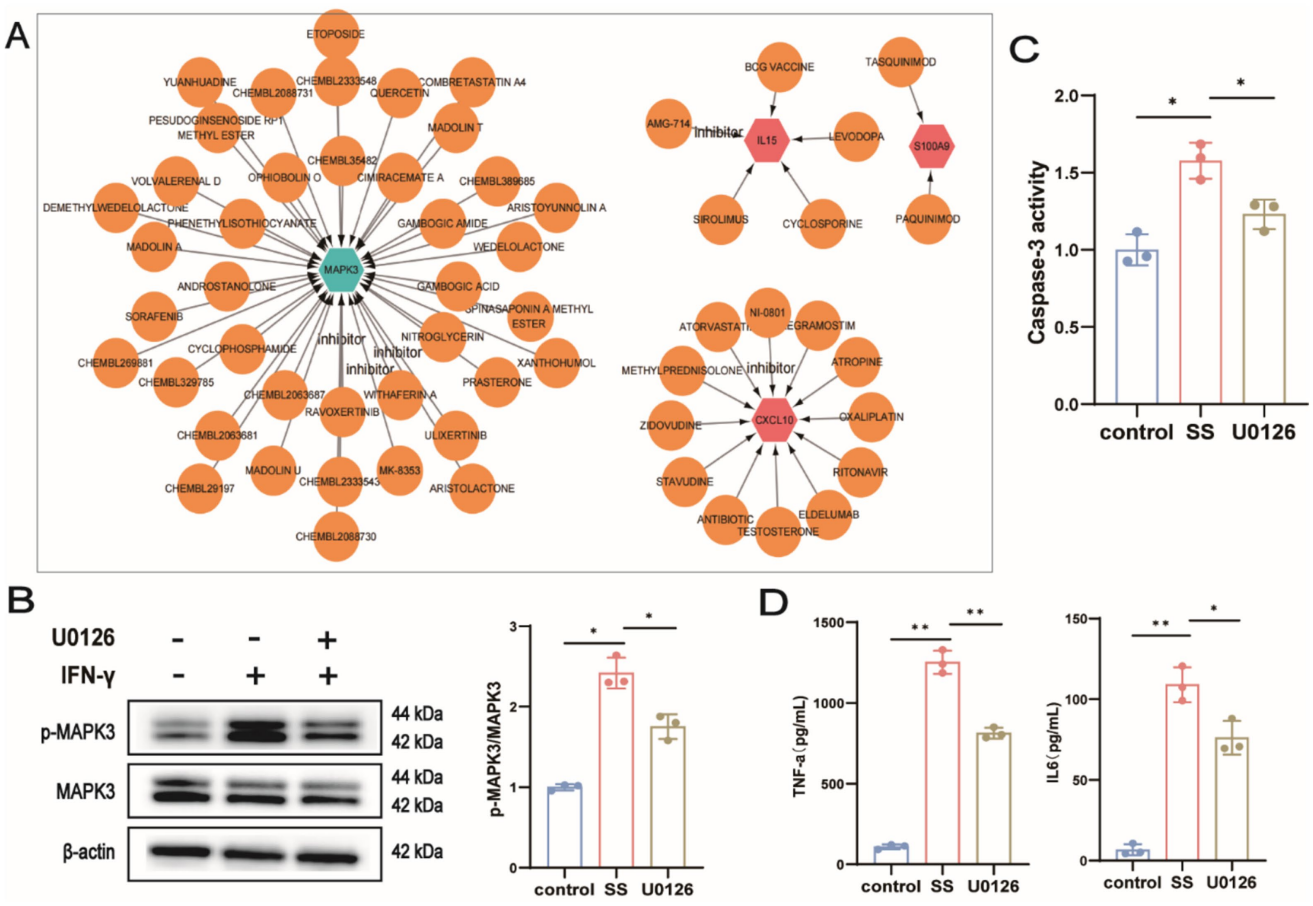
**(A)** Drug-gene interaction network. Red nodes represent upregulated genes, green nodes downregulated genes, and brown nodes indicate predicted drugs; **(B)** WB analysis of MAPK3; **(C)** Caspase-3 activity assay; **(D)** ELISA measurement of TNF-*α* and IL6 levels in cell supernatants. ^ns^*p* > 0.05, *p < 0.05, **p < 0.01, ***p < 0.001, ****p < 0.0001.

The DGIdb online database was used to identify potential therapeutic drugs targeting *MAPK3, CXCL10, IL15, IFI27,* and *S100A9* (Figure 12A). The search yielded 59 candidate drugs for SS treatment, including 40 targeting *MAPK3*, 12 targeting *CXCL10*, 5 targeting *IL15*, and 2 targeting *S100A9*. No drugs were identified targeting IFI27 were identified. Subsequently, an *in vitro* SS inflammation model was constructed by stimulating SMG-C6 cells with 50 ng/mL IFN-*γ* for 24 h (18) to investigate the effect of the MAPK3 inhibitor U0126. Results demonstrated that IFN-γ stimulation significantly increased caspase-3 levels, activated the MAPK3 signaling pathway, and elevated levels of inflammatory cytokines TNF-*α* and IL6. Conversely, treatment with U0126 markedly decreased apoptosis and attenuated inflammation (Figures 12B–D), further confirming the potential of MAPK3 as a biomarker for SS.

## 4 Discussion

SS is a chronic autoimmune disease characterized by dryness symptoms experienced by over 95% of patients. This glandular dysfunction may persist for over a decade, significantly impairing patients’ quality of life. Additionally, SS has been described as an autoimmune epithelitis, underscoring the crucial role of epithelial cells in maintaining normal glandular function (18). Immune-mediated damage to salivary gland epithelial cells in SS reduces saliva secretion. Various cell death mechanisms involving epithelial cells and neutrophils contribute to glandular damage in SS, among which anoikis plays a central role. SS-related salivary glands exhibit prominent morphological and functional changes in acinar and ductal structures, accompanied by extensive ECM remodeling. Normal cellular survival depends on adhesion to the ECM, which is essential for tissue structure and function, particularly in epithelial tissues. Loss of adhesive interactions between epithelial cells and the ECM can alter gene expression, frequently triggering anoikis. Consequently, detachment-induced epithelial cell death of (anoikis) significantly contributes to glandular dysfunction in SS (19). Studies suggest that anoikis-driven apoptosis of human salivary gland epithelial cells, resulting from ECM detachment or inappropriate ECM interactions, significantly promotes SS pathology. Furthermore, dysregulated expression of the ECM protein fibulin-6 in salivary gland epithelial cells may also influence SS initiation and progression (20). In this study, we employed three machine learning algorithms to examine the role of ARGs in SS and explored the potential contribution of anoikis to disease progression.

Initially, we identified 35 ARGs significantly differentially expressed between SS and healthy controls. Subsequent GO and KEGG enrichment analyses revealed these genes to be primarily involved in disease-related signaling pathways, including influenza A, coronavirus disease (COVID-19), hepatitis C, measles, pertussis, toxoplasmosis, and rheumatoid arthritis. Additionally, these genes were closely associated with pathways linked to inflammation, viral infections, and necroptosis, such as the NLR signaling pathway, TLR signaling pathway, Epstein–Barr virus (EBV) infection, chemokine signaling pathway, IL-17 signaling pathway, TNF signaling pathway, and necroptosis. The NLR and TLR signaling pathways, central components of innate immune responses, have been extensively studied in SS. Both NLRs and TLRs are pattern recognition receptors (PRRs) that initiate immune responses upon stimulation. NLRP3, a critical inflammasome within the NLR family, mediates innate immunity against microbial and environmental triggers. The NLRP3, inflammasome is several implicated in multiple autoimmune diseases, including systemic lupus erythematosus (21), SS (22) and rheumatoid arthritis (23). SS patients exhibit excessive activation of monocytic and macrophagic inflammasomes, notably NLRP3 (22), Moreover, along with the activation of the NLRP3 inflammasome and subsequent IL-18 secretion in salivary glands significantly enhance innate immune responses (24). Activation of NLRP3 also promotes substantial production of inflammatory cytokines IL-1β and IL-18 in peripheral blood stem mononuclear cells (PBMCs), driving chronic inflammation and tissue injury in SS (25). Similarly, TLRs are PRRs that recognize nucleic acids to induce type I interferon (IFN) production. Recent RNA sequencing studies comparing SS and healthy salivary glands underscore the critical involvement of TLR signaling in SS pathology (26). Specifically, TLR7 expression in ductal epithelial cells increases the cytoplasmic autoantigen Ro52, leading to epithelial damage in SS glands (27). Zhang et al. (28) the reported that TLR signaling is crucial in SS-associated thrombocytopenia, activating the MyD88/NF-κB pathway and subsequently promoting inflammatory cytokine production, including TNF-*α* and IL-6. Targeting in pivotal molecules within the TLR pathway, such as TLR7 or MyD88, represents a promising therapeutic strategy for SS. In addition to inflammatory signaling, viral infections, particularly EBV and hepatitis C virus, have been strongly associated with SS and recognized as clinical risk factors. Elevated EBV DNA levels have been detected in salivary glands and PBMCs of SS patients; however, the exact molecular mechanisms underlying EBV-related SS remain incompletely understood (29).

In recent years, machine learning approaches have been increasingly utilized in SS diagnosis, identification of critical biomarkers, and immune-cell characterization due to their superior predictive performance, reduced error rates, and enhanced reliability. From an initial selection of 35 candidate genes, 14 feature genes were ultimately identified as potential biomarkers for SS: *MAPK3, NAT1, CXCL10, IL15, PRDX4, BIRC3, EZH2, SKI, MAD2L1, ATP2A3, HMGA1, BST2, IFI27,* and *S100A9.* Apart from *ATP2A3, HMGA1, MAPK3,* and *SKI,* the remaining genes exhibited elevated expression predominantly in SS patients. Among these, *NAT1, BIRC3, EZH2, MAD2L1, ATP2A3, HMGA1,* and *BST2* are known cancer-associated genes. *NAT1* is implicated in breast cancer (30), *BIRC3* associated with such as colorectal cancer (31), liver cancer (32), and chronic lymphocytic leukemia (33), *EZH2* shows overexpression in lung adenocarcinoma (34), endometrial cancer (35), and prostate cancer (36), *MAD2L1* is related to cholangiocarcinoma (37), lung adenocarcinoma (38), and breast cancer (39), *ATP2A3* is a characteristic marker for bladder cancer (40) and head and neck squamous cell carcinoma (41), *HMGA1* is involved in of lung adenocarcinoma (42) and breast cancer (43), and *BST2* is a key gene in pancreatic cancer (44) and oral squamous cell carcinoma (45).

Collectively, these findings suggest a possible connection between tumorigenesis and SS. Although explicit evidence demonstrating this association remains limited, these genes merit further investigation for their potential roles in SS pathogenesis. *SKI* and *PRDX4*, associated with multiple inflammatory diseases (46, 47), have unclear roles in SS and were therefore not the focus of this study. Instead, emphasis was placed on *MAPK3, IL15, S100A9, IFI27,* and *CXCL10*. MAPK3, a critical member of the MAPK family, participates in numerous fundamental cellular processes, including cell proliferation, differentiation, apoptosis, and inflammatory responses. Many studies have demonstrated its role in regulating B cell functions via the MAPK3 (Erk1/2) signaling pathway (48) and modulating inflammatory cytokine production (49). Activation of the MAPK signaling pathway is known to facilitate excessive T and B cell proliferation and activity, thereby exacerbating SS progression (50). Previous reports have shown that inhibiting MAPK signaling alleviates glandular symptoms in SS (50). Additionally, IL-17 stimulation of salivary gland epithelial cells (SGECs) activates the MAPK3 pathway, phosphorylating Erk1/2 and subsequently promoting glandular inflammation, epithelial-mesenchymal transition (EMT), fibrosis, and glandular dysfunction (51). Similar findings confirmed increased phosphorylation of MAPK3 in salivary glands of SS patients (52). Intriguingly, recent studies reported MAPK3 as an upstream regulator of mTOR, a core autophagy gene, activating mTOR signaling and consequently suppressing autophagy (53). This finding raises the question of whether MAPK3 may, to some extent, alleviate excessive autophagy and cell death, thereby positively influencing glandular function. Future studies should explore this pathway further to elucidate its precise role in SS pathogenesis. In agreement with these findings, our animal experiments revealed enhanced phosphorylation (activation) of MAPK3 protein despite decreased MAPK3 mRNA expression, possibly related to differences between total mRNA levels and protein phosphorylation status. IL15, a key regulatory cytokine predominantly produced by macrophages and non-lymphoid cells, signals via JAK/STAT and Ras/MAPK pathways and modulates cell survival through balancing pro-apoptotic and anti-apoptotic signals in the PI3K pathway. Elevated IL15 mRNA and protein levels have been documented in the salivary glands of SS patients, promoting glandular T and B cell activation and persistent inflammation (54). Moreover, IL15 expression was markedly upregulated in acinar and ductal cells, indicating its potential as a therapeutic target for SS-associated inflammation (54), consistent with our experimental results. S100A9, a pro-inflammatory protein implicated in various inflammatory disorders, is recognized as a promising biomarker for SS. Its expression in saliva is significantly elevated in SS patients compared with healthy controls, indicating its potential as an early diagnostic marker for SS (55). Additionally, elevated S100A9 expression in PBMCs from SS patients promotes pro-inflammatory cytokine production (56). Together with our experimental findings, these results underscore the considerable diagnostic potential of S100A9 in SS, warranting further exploration as a specific biomarker. Activation of the IFN pathway is a hallmark immunological feature of SS (57), with approximately two-thirds of SS patients displaying increased IFN activity (58). Both IFI27 and CXCL10 are interferon-induced proteins (59, 60). IFN pathway activation, critical in immune responses against bacterial and viral infections, stimulates innate immunity through recognition of pathogenic agents (61). Specifically, type I IFN induces B cell proliferation and differentiation via the TLR7 signaling pathway, facilitating BAFF production (61), enhancing B cell activation, and promoting adaptive immunity. Thus, IFN pathway activation represents a critical junction linking innate and adaptive immune responses in SS and constitutes a promising therapeutic target for the disease.

To enhance our understanding of SS pathogenesis, we comprehensively analyzed immune cell infiltration in SS using the CIBERSORT algorithm. Our results revealed increased levels of memory B cells, activated memory CD4 + T cells, gamma delta T cells, M1 macrophages, M2 macrophages, and activated dendritic cells in SS tissues. Conversely, naive CD4 + T cells, regulatory T cells (Tregs), and resting NK cells exhibited decreased abundance. It is well-established that significant activation of both T and B cells occurs in the salivary glands and peripheral blood of SS patients (62), and substantial research supports these observations. One study employing single-cell sequencing extensively characterized peripheral blood B cell subsets in SS patients, underscoring the critical role of B cells in SS pathogenesis and emphasizing the need for further exploration of B cell subset-specific contributions to SS (63). Increased tissue-resident memory B cells may elevate the risk of lymphoma development in SS patients (64). Activation of B cells, however, requires T-cell involvement; notably, CD4 + T cells and B cells predominate the inflammatory infiltrates observed in salivary and lacrimal glands (65, 66). Additionally, dendritic cells, as the primary source of IFN production, are highly expressed in SS (65), consistent with our findings related to IFN pathway activation. Furthermore, published single-cell analyses have confirmed elevated infiltration of these immune cell populations in SS patients and animal models (7, 67–69). Collectively, these immune cells contribute to glandular inflammatory infiltration by releasing inflammatory cytokines, potentially interacting with glandular epithelial cells and compromising gland structure and function. Thus, SS pathogenesis likely results from complex interactions among diverse immune and tissue-resident cells rather than a single cell type.

Following the ceRNA hypothesis, we constructed a ceRNA network containing five key genes, 22 miRNAs, and 57 lncRNAs. Using the MCC algorithm, we identified 10 key miRNAs: miR-214-3p, miR-30b-3p, miR-590-3p, miR-542-3p, miR-148a-3p, miR-130a-3p, miR-483-5p, miR-486-3p, miR-452-3p, and miR-296-5p. Among these, only five (miR-30b-5p, miR-148a-3p, miR-130a, miR-483-5p, and miR-486-3p) have been investigated in relation to SS; thus, we focused our discussion on these miRNAs. It is well-established that miRNAs circulate stably and reproducibly in serum and plasma, making them promising biomarkers for various diseases. miR-30b-5p is significantly dysregulated in minor salivary glands from SS patients (70). Another study reported substantially downregulated miR-30b-3p expression in SS (71, 72), negatively correlating with BAFF levels in B cells from SS patients. Furthermore, inhibition of miR-30b-5p in THP-1 cells markedly elevated BAFF expression, suggesting that reduced miR-30b-5p may enhance BAFF-mediated inflammation in SS patients (72). Additionally, decreased miR-148a expression was reported in Treg-deficient mice, a recognized autoimmune disease model, with ROC analysis indicating its potential utility as a diagnostic biomarker (73). However, direct evidence regarding the sensitivity and specificity of miR-148a for SS diagnosis is currently lacking. Meanwhile, miR-130a was consistently downregulated in conventional dendritic cells from SS patients, leading to increased MSK1 expression and subsequent production of inflammatory cytokines (e.g., IL-12 and 12, TNF-*α*), thereby exacerbating salivary gland inflammation (74). Targeting miR-130a to inhibit MSK1 and inflammatory cytokine release may thus represent a novel therapeutic approach for SS (74). Moreover, miR-483-5p expression is significantly upregulated in PBMCs from SS patients, and particularly in minor salivary glands of anti-Ro/SSA and anti-La/SSB double-positive SS patients compared with seronegative counterparts (75). Conversely, Eleni et al. found no significant difference in serum miR-483-5p levels between healthy controls and SS patients, although miR-483-5p was specifically elevated in localized scleroderma and systemic sclerosis (SSc), indicating its potential role as a biomarker for SSc (76). Finally, although our analysis identified miR-486-3p as a possible SS biomarker, previous studies reported differential miR-486-3p expression in salivary glands as a potential marker for distinguishing feature between IgG4-related disease and SS, suggesting the exact role of miR-486-3p in SS remains uncertain (77). These collective insights may contribute substantially to understanding the molecular mechanisms driving SS progression.

Furthermore, we identified 59 candidate drugs from the DGIdb database for potential SS treatment, subsequently narrowing this list to eight therapeutic agents: methylprednisolone, cyclophosphamide, cyclosporine, atorvastatin, etoposide, sirolimus, paquinimod, and quercetin. Interestingly, methylprednisolone, cyclophosphamide, and cyclosporine have already been widely used clinically in SS, particularly during high disease activity or systemic involvement. According to the 2020 EULAR guidelines for SS management, glucocorticoids, particularly methylprednisolone, are recommended for controlling active systemic involvement, with an emphasis on using the lowest effective dose for the shortest possible duration. Immunosuppressants such as cyclophosphamide and cyclosporine may serve as alternatives to glucocorticoids, although a definitive consensus regarding their use has yet to be established (78). Cyclophosphamide has shown efficacy in alleviating interstitial lung disease symptoms associated with connective tissue disorders and is recommended for SS patients with chronic tubulointerstitial nephritis, particularly those presenting with high IgG levels and renal function impairment (79, 80). Atorvastatin and etoposide, known primarily as widely utilized in other clinical disciplines, may exhibit unexpected therapeutic potential for SS patients with concurrent conditions. For example, atorvastatin, a lipid-lowering agent, has demonstrated anti-inflammatory effects in SS by inhibiting IL-1β, PGE2, and MMP-3 production in rat submandibular glands (81). Etoposide, an anticancer drug, has been reported to selectively eliminate pathologically activated T lymphocytes and effectively suppress inflammatory cytokine release in cases of SS complicated by macrophage activation syndrome. However, these findings are based on limited case reports, lacking extensive clinical or fundamental research support (82). Apart from medications already clinically available, we identified three additional small-molecule drugs with potential therapeutic effects on SS. These agents have undergone limited validation in preclinical or clinical studies, yet may provide promising opportunities for future SS drug development. Sirolimus (rapamycin), an mTOR inhibitor, has demonstrated clinical efficacy in treating SS-associated thrombocytopenia (83). Mechanistically, it suppresses T follicular helper (Tfh) cell differentiation by reducing mTOR activity, maintaining a balance between Tfh and T follicular regulatory (Tfr) cells (84). Additionally, sirolimus reduces glandular inflammation and improves tear production (85). Paquinimod, an alarmin inhibitor, inhibits alarmin expression in an IL14α transgenic mouse model of SS, promoting Ca2 + influx, restoring salivary gland function, reducing immune cell infiltration, and preventing SS progression (86). Finally, we discovered quercetin, a natural compound, as a potential therapeutic agent for SS. Quercetin mitigates salivary gland cell apoptosis and inflammation by inhibiting the LP/OB-R/JAK2/STAT3 signaling pathway, thereby providing new opportunities for alleviating dry-mouth symptoms in SS (87). Currently, various novel drugs have yielded encouraging outcomes in SS treatment; nevertheless, several challenges remain regarding their clinical translation. First, addressing potential drug toxicity during research and development poses significant difficulties. Structural optimization or modification of novel drugs to enhance therapeutic effects while reducing toxicity is crucial. Adjusting administration routes or employing advanced technologies, such as nanocarrier delivery systems, could achieve targeted drug delivery, enhancing efficacy and reducing side effects. Furthermore, translating preclinical findings to clinical practice represents the most challenging step, requiring extensive and rigorous experimentation. Finally, exploring traditional Chinese medicine as adjunctive therapy to enhance the therapeutic effects of essential yet potentially toxic medications could present an intriguing area for future research.

In summary, our study identified characteristic genes associated with SS and anoikis, employing enrichment analyses to elucidate their biological roles. We highlighted several pathogenic mechanisms potentially mediated by key hub genes. Utilizing three machine-learning algorithms, we identified five essential genes serving as biomarkers, likely involved in regulating the SS immune microenvironment. Based on these findings, we constructed a ceRNA network and predicted candidate therapeutic drugs, thus providing novel insights into SS treatment. However, our study has limitations, primarily due to reliance solely on data analysis without sufficient experimental validation. Therefore, further animal studies are necessary to substantiate our conclusions.

## Data Availability

The original contributions presented in the study are included in the article/Supplementary material, further inquiries can be directed to the corresponding author/s.
