# Correction: Identification of anoikis-related genes and immune infiltration characteristics in Sjögren's syndrome based on machine learning

**DOI:** 10.3389/fmed.2025.1741480

**Published:** 2025-11-19

**Authors:** Lei Wang, Ziqi Xu, Xinpeng Zhou, Ying Liu, Mengjie Wang

**Affiliations:** 1 Department of Rheumatology, Affiliated Hospital of Shandong University of Traditional Chinese Medicine, Jinan, China; 2 Institute of Pharmacy, Shandong University of Traditional Chinese Medicine, Jinan, China

**Keywords:** Sjögren's syndrome, anoikis, machine learning, ceRNA network, immune infiltration

Author [Ziqi Xu] was erroneously spelled as [Ziqi Zhou].

In a published article, there is an error in the author name list. The second author is shown as “Ziqi Zhou”. Correctly expressed as “Ziqi Xu”.

The original version of this article has been updated.

